# Predictive Modeling of Heterogeneous Treatment Effects in RCTs: A Scoping Review

**DOI:** 10.1001/jamanetworkopen.2025.22390

**Published:** 2025-07-22

**Authors:** Joe V. Selby, Carolien C. H. M. Maas, Bruce H. Fireman, David M. Kent

**Affiliations:** 1 Division of Research, Kaiser Permanente Northern California, Pleasanton; 2 Tufts Predictive Analytics and Comparative Effectiveness Center, Tufts University School of Medicine, Boston, Massachusetts; 3 Department of Public Health, Erasmus University Medical Center, Rotterdam, the Netherlands; 4 Division of Research, Kaiser Permanente Northern California, Pleasanton

## Abstract

**Importance:**

The Predictive Approaches to Treatment Effect Heterogeneity (PATH) Statement of 2020 proposed predictive modeling for identifying heterogeneity in treatment effects (HTE) in randomized clinical trials (RCTs). It described 2 approaches: risk modeling, which develops a multivariable model predicting individual baseline risk of study outcomes and then examines treatment effects across strata of predicted risk, and effect modeling, which develops a model that directly predicts individual treatment effects using a variety of regression and machine learning methods.

**Objective:**

To identify, describe, and evaluate findings from reports that cited the PATH Statement and presented predictive modeling of HTE in RCTs.

**Evidence Review:**

Reports were identified using PubMed, Google Scholar, Web of Science, and SCOPUS through July 5, 2024. Using double review with adjudication, reports were assessed for consistency with PATH Statement recommendations, credibility of HTE findings (applying criteria adapted from the Instrument to Assess Credibility of Effect Modification Analyses), and clinical importance of credible findings.

**Findings:**

A total of 65 reports (presenting 31 risk models and 41 effect models) analyzing 162 RCTs were identified, with credible, clinically important HTE in 24 reports (37%). Contrary to PATH Statement recommendations, only 25 of 48 studies with positive overall findings included a risk model. Most effect models were exploratory, including multiple predictors with little prior evidence for HTE. Claims of HTE were noted in 23 risk modeling and 31 effect modeling reports but were more likely to meet credibility criteria with risk modeling (20 of 23 reports [87%]) than effect modeling (10 of 31 reports [32%]). For effect modeling, validation of HTE findings in external datasets was critical in establishing credibility. Credible HTE from either approach was usually judged clinically important (24 of 30 reports [80%]). In the 19 reports from RCTs suggesting overall treatment benefits, modeling identified subgroups of 5% to 67% of patients predicted to experience no benefit or net treatment harm. In the 5 reports that found no overall benefit, subgroups of 25% to 60% of patients were nevertheless predicted to benefit.

**Conclusions and Relevance:**

This scoping review of 65 reports of multivariable predictive modeling of HTE in RCTs identified credible, clinically important HTE in 37%. Risk modeling was more likely than effect modeling to find credible HTE, but external validation of HTE findings served to increase the credibility of findings from exploratory effect models.

## Key Points

**Question:**

How often has the 2020 Predictive Approaches to Treatment Heterogeneity (PATH) Statement been cited in published predictive models from randomized clinical trials (RCTs), how well have these reports adhered to PATH Statement recommendations, and how credible are their findings of heterogeneity of treatment effects (HTE)?

**Findings:**

In this scoping review of 65 reports of predictive models analyzing 162 RCTs, more than one-third (24 reports) identified credible, clinically important HTE. Risk-based modeling was more likely than effect modeling to meet criteria for credibility (87% vs 32%).

**Meaning:**

The findings of this scoping review illustrate the value of predictive modeling of HTE for making RCT results more useful and suggest that external validation in independent patient cohorts can greatly increase credibility, especially for effect models.

## Teaser

This scoping review describes and evaluates findings from reports that cited the Predictive Approaches to Treatment Effect Heterogeneity (PATH) Statement and presented predictive modeling of heterogeneity in treatment effects in randomized clinical trials (RCTs).

## Introduction

Overall, or average, treatment effects from randomized clinical trials (RCTs) provide limited information to patients making personal treatment decisions.^1,2,3,4,5,6^ Even in strongly positive RCTs, some patients do not benefit from the favored treatment and yet may experience adverse effects. In negative trials, some patients may nevertheless experience benefit with the treatment under study. Patients and clinicians would benefit greatly if more individualized evidence could be generated and reported from RCTs.

Most RCT reports continue to limit examination for possible heterogeneity of treatment effects (HTE) to one-at-a-time comparisons between numerous patient subgroups, eg, men vs women, persons with vs without diabetes, even though guidelines for identifying HTE in RCTs^1,7,8,9,10,11^ have long emphasized that such analyses are at great risk for both false-positive and false-negative findings. An additional limitation of this approach is that individuals simultaneously belong to multiple subgroups that may vary in whether or how they benefit. Thus, guidelines consistently recommend limiting the number of subgroups studied to those with prior evidence or strong biologic or clinical rationale for HTE and using caution in interpreting or applying findings to clinical practice.

The emergence of precision medicine^12^ and patient-centered outcomes research^13^ heightens interest in identifying important HTE. In 2020, an expert panel funded by the Patient-Centered Outcomes Research Institute published the Predictive Approaches to Treatment Effect Heterogeneity (PATH) Statement,^14,15^ which described predictive modeling approaches that incorporate multiple patient attributes simultaneously to identify HTE and predict individualized treatment effects. The PATH Statement pointed out that HTE, sometimes referred to as *treatment effect modification* and tested as a statistical interaction, should be sought on both the absolute scale (eg, as risk differences) and on the relative scale (eg, as ratios of risks, odds, or hazards) and emphasized that absolute treatment effects matter more to individual patients and clinicians making treatment decisions.

The PATH Statement distinguished 2 approaches to predictive modeling. Risk modeling incorporates multiple baseline patient characteristics into a model predicting risk for the RCT’s outcome (usually the primary outcome). In a second step, both absolute and relative treatment effects are examined across prespecified strata (eg, quarters) of predicted risk.^16^ In the second approach, effect modeling, individual treatment effects are directly estimated, using either regression methods that incorporate treatment, multiple covariates and interaction terms, or, more recently, a variety of more flexible, nonparametric, data-driven machine learning algorithms.^17,18,19,20^

The PATH Statement recommended risk modeling whenever an RCT demonstrates an overall treatment effect. Risk of study outcomes varies substantially in clinical practice and in most RCT populations, and the absolute benefit from an effective treatment is expected to increase as baseline risk increases,^21,22^ a mathematical relationship that has been called *risk magnification*.^23^ This relationship is often referenced in the evidence-based medicine literature^10,24,25^ and is implicit in clinical practice and in guidelines that reserve costly or riskier treatments for patients with higher baseline risk.^26,27^ The PATH Statement recommended use of validated external models for predicting risk if available, but cited evidence that in their absence, valid models can be developed within the RCT population, using baseline covariates and observed study outcomes from both study arms.^28,29^ The PATH Statement pointed out that risk modeling would be unlikely to identify HTE when overall RCT results are null, where both a clinical rationale and the mathematical expectation of risk magnification are lacking. More generally, the PATH Statement urged caution in conducting any HTE analyses when overall results are null.

Effect modeling permits a more robust examination of possible HTE. The PATH Statement recommended its use when there are previously established or strongly suspected effect modifiers. However, it emphasized the vulnerability of effect modeling approaches to over-fitting^29^ and false-positive findings when multiple potential interactions are considered and recommended use of statistical methods that aim to reduce over-fitting of data. It additionally suggested validation of effect model findings in external datasets when possible.

We conducted a scoping review^29^ to describe the frequency with which predictive modeling analyses of RCT data have appeared and cited the PATH Statement since its publication and the consistency of analyses with PATH Statement consensus criteria (eTable 1 in Supplement 1). We also assessed the frequency of claimed HTE and the credibility and clinical importance of these claims. We used criteria adapted from the Instrument to Assess Credibility of Effect Modification Analyses (ICEMAN),^11^ and when HTE was found to be credible, we applied the PATH Statement definition of clinical importance: “variation in the risk difference across patient subgroups potentially sufficient to span clinically-defined decision thresholds,”^14^ ie, supporting differing treatment recommendations for patient subgroups.

## Methods

### Identification of Reports Eligible for Inclusion

In conducting this scoping review, we followed the recommendations of the Preferred Reporting Items for Systematic Reviews and Meta-analyses Extension for Scoping Reviews (PRISMA-ScR) reporting guideline.^30^ We used the Cited By functions in PubMed, Google Scholar, Web of Science, and the SCOPUS database (eTable 2 in Supplement 1) to identify reports that appeared between January 7, 2020 and July 24, 2024, cited the PATH Statement, and presented multivariable predictive modeling in RCT data to identify HTE. We included non–peer-reviewed reports from preprint archives and dissertations on institutional websites.

**Figure 1.**
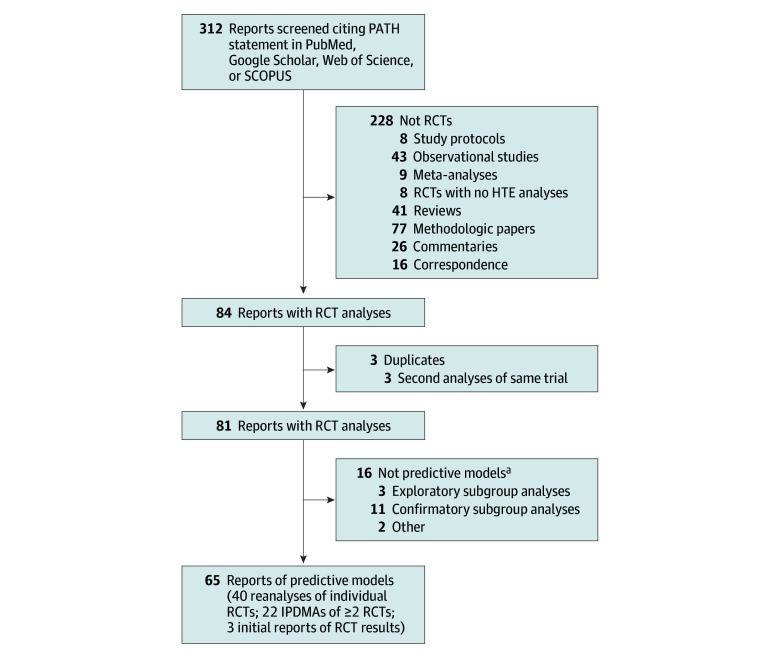
Flowchart for Identification and Screening of Reports All reports considered cited the Predictive Approaches to Treatment effect Heterogeneity (PATH) Statement. Reports were excluded if they did not present a predictive model of individual treatment effects from randomized clinical trial (RCT) data. IPDMA indicates independent patient data meta-analysis. ^a^Details of these 16 studies are presented in eTable 2 in Supplement 1.

Among 312 citations identified (Figure 1), 83^31,32,33,34,35,36,37,38,39,40,41,42,43,44,45,46,47,48,49,50,51,52,53,54,55,56,57,58,59,60,61,62,63,64,65,66,67,68,69,70,71,72,73,74,75,76,77,78,79,80,81,82,83,84,85,86,87,88,89,90,91,92,93,94,95,96,97,98,99,100,101,102,103,104,105,106,107,108,109,110,111,112,113^ involved analyses of data from RCTs. Sixteen studies^31,32,33,34,35,36,37,38,39,40,41,42,43,44,45,46^ (eTable 2 in Supplement 1) were excluded because they did not use multivariable predictive models. Three analyses were represented by 2 reports each^47,48,49,50,51,52^ and 1 report^111^ presented predictive models from 2 distinct trials in different clinical areas, leaving 65 reports analyzing data from 162 RCTs.

### Review of Predictive Model Reports

Variables collected and coding instructions are presented in eTable 3 and eTable 4 in Supplement 1, respectively. All details of analytic strategies and findings of HTE were doubly reviewed by J.V.S. and 1 coauthor. A learning set of 6 reports was reviewed and discussed by all coauthors. Thereafter, coreviewers discussed and adjudicated initial disagreements. Adjudicated data were stored in a master spreadsheet (Microsoft Excel, version 2501) and imported to Stata software version 18.5 (StataCorp) for summarization.

Reviewers classified each report as risk modeling, effect modeling, or both and further classified effect models into those based primarily on regression methods (eg, ordinary least squares, logistic, proportional hazards, bayesian regression methods) and those using more flexible data-driven machine learning algorithms. For both risk and effect modeling analyses, we assessed consistency with PATH Statement Consensus Criteria (eTable 1 in Supplement 1), whether authors reported having found HTE on either absolute or relative scales, and whether results of statistical testing for HTE were presented. We included results of statistical tests for treatment × covariate interactions from regression models, direct contrasts of treatment effects across subgroups, CIs for subgroup treatment effect estimates, and overall tests of the null hypothesis of homogeneity of treatment effect in machine learning algorithms. We determined whether the performance of final models for predicting individual or subgroup treatment effects was validated in datasets external to the derivation population, including validations conducted in entirely distinct RCTs, those conducted in prespecified, nonrandom subsets of the original RCT population (eg, subsets selected on bases of geography [trial sites] or time of enrollment), and those conducted in large observational cohorts.

### Statistical Analysis

To assess credibility of claimed HTE, on either absolute or relative scales, we adapted 4 of the 5 ICEMAN criteria for RCTs.^11,114^ Detailed description and scoring guidance for each criterion and the overall credibility score are provided in eTable 5 in Supplement 1. Although ICEMAN criteria were originally developed for evaluating treatment effect modification by single covariates, 4 apply readily to predictive modeling with multiple covariates. These include (1) did the authors test only a small number of interactions; (2) was possible effect modification by each covariate supported by prior evidence; (3) if the covariate was a continuous variable, were arbitrary, data-driven cut points avoided; and (4) does a statistical test for interaction suggest that chance is an unlikely explanation of the apparent HTE? The fifth criterion, whether the direction of interaction was hypothesized in advance, is not applicable to predictive modeling, given that multiple covariates and potentially complex interactions are evaluated simultaneously. No single criterion, including that of statistical testing, is treated as either sufficient or necessary for establishing overall credibility. Possible overall credibility scores range from 1 to 4 (very low, low, moderate, or high credibility).

Risk modeling can be expected to score well when ICEMAN criteria are applied because it considers a single effect modifier (the baseline risk score), has strong prior theoretical and empirical support^21^ for HTE, at least on the absolute scale, and uses prespecified rather than data-driven risk score cut points. Most effect models tested multiple potential effect modifiers, often with little prior evidence, and frequently used data-driven cut points, and therefore would be expected to score poorly. However, we gave considerable weight to validation of effect model predictions in an external population. External validation refers to an assessment of whether the predicted individual treatment effects correspond to observed treatment effects when applied to patients from a population not used in model development. External validation evaluates a single potential effect modifier (the effect score) which has prior evidence for HTE and prespecified cut points.^115^ Overall credibility usually rose to moderate if models performed well in external validation, even if credibility of the derivation analyses would have scored very low.

We classified all reports scored as at least moderate overall credibility as credible and assessed findings for clinical importance. Per the PATH Statement, clinical importance is based on the size and direction of observed differences in absolute treatment effects between subgroups and whether these differences appear sufficient to support differing treatment recommendations. An additional consideration was whether findings for all outcomes studied, including adverse effects of treatment, were consistent in supporting the same treatment choice.

## Results

### General Description

Predictive models of HTE appeared with increasing frequency each year following publication of the PATH Statement (Table 1). Among the 65 reports included,^47,48,49,50,51,52,53,54,55,56,57,58,59,60,61,62,63,64,65,66,67,68,69,70,71,72,73,74,75,76,77,78,79,80,81,82,83,84,85,86,87,88,89,90,91,92,93,94,95,96,97,98,99,100,101,102,103,104,105,106,107,108,109,110,111,112,113^ we identified 31 risk modeling and 41 effect modeling analyses. Seven reports^61,85,87,92,106,110,112^ presented both risk and effect modeling analyses. Most effect modeling reports considered large numbers of potential effect modifiers, and most used data-driven, nonparametric analytic methods.

**Table 1.** Characteristics of the 72 Analyses (65 Reports) of Predictive Models for Possible HTE^a^

Characteristic	Modeling analyses, No. (%)	
	Risk (n = 31)	Effect (n = 41)
Publication year		
2020	2 (6)	3 (7)
2021	4 (13)	8 (20)
2022	7 (23)	7 (17)
2023	9 (29)	15 (37)
2024 (through July 5)	9 (29)	8 (20)
Initial publication status		
Peer-reviewed	24 (77)	34 (83)
Preprint archive^b^	3 (10)	7 (17)
Dissertation	4 (13)	1 (2)
Data source		
Reanalyses of single RCT	16 (52)	26 (63)
Initial analyses of RCT(s)	2 (6)	0
IPDMA of ≥2 RCTs	13 (42)	15 (37)
Comparative effectiveness research^c^		
Yes	13 (42)	25 (61)
No	18 (58)	16 (39)
Overall RCT results^d^		
Null (no overall difference)	6 (19)	12 (29)
Modest effect	7 (23)	13 (32)
Strong effect	18 (58)	16 (39)
Sample size of HTE analyses, median (IQR) [range], No. of patients	1907 (999-3740) [574-330 460]	2294 (1250-8828) [200-26 877]
Type of risk model used (n = 31)		
External model applied	14 (45)	NA
Internal model developed	17 (55)	NA
No. of potential effect modifiers tested in effect models, median (IQR) [range] (n = 41)	NA	17 (10-22) [2-58]
Type of effect models employed		
Regression methods (n = 13)^e^		
Conventional regression^f^	NA	6 (46)
Regression with penalization to avoid over-fitting^g^	NA	7 (54)
Machine learning algorithms (n = 28)^h^		
Causal forests	NA	13 (46)
Other tree-based methods^i^	NA	10 (36)
Meta-learner methods	NA	9 (32)

Abbreviations: HTE, heterogeneity of treatment effects; IPDMA, independent patient data meta-analysis; NA, not applicable; RCT, randomized clinical trial.

^a^ Seven reports^61,85,87,92,106,110,112^ presented both risk and effect modeling analyses.

^b^ Nine of 10 reports^50,59,71,74,77,82,89,103,112^ originally identified through preprint archives have subsequently been published in peer-reviewed journals. One report^90^ has not appeared as a peer-reviewed publication.

^c^ Comparative effectiveness was defined as comparison of 2 or more alternative, active interventions (eTable 4 in Supplement 1).

^d^ Null was defined as overall treatment effect does not differ significantly from zero; modest effect, estimated relative risk reduction is 20% or less; strong effect, estimated relative risk reduction greater than 20%. For continuous outcomes, a standardized mean difference significantly greater than 0, but no greater than 0.8 was considered moderate; and a standardized mean difference greater than 0.8 was considered strong.

^e^ These include only reports based solely on regression models without additional analyses using more flexible machine learning algorithms.

^f^ Conventional regression includes linear, logistic, and Cox proportional hazards regression models that did not use either penalization or regularization methods (eg, least absolute shrinkage and selection operator, penalized ridge regression, elastic net) or cross-validation methods explicitly intended to reduce over-fitting.

^g^ These include regression models that incorporated penalization or regularization methods or cross-validation methods explicitly intended to reduce over-fitting (or both).

^h^ Because some reports featured more than 1 machine learning algorithm, these items are not mutually exclusive.

^i^ Includes examples of model-based recursive partitioning, gradient-boosted regression trees, random forest, and bayesian additive regression trees.

### Reviewer Agreement

Excluding 6 reports (presenting 6 effect models and 1 risk model) used for training reviewers, initial between-reviewer disagreement rates for 19 doubly reviewed items ranged from 0% to 47%, with an overall mean of 10.1% (eTable 3 in Supplement 1). Initial disagreement was greater for assessments of the credibility and clinical importance of claimed HTE, possibly because assessment of these items was added near the end of data collection and without additional training. Nevertheless, such disagreements were easily resolved on discussion.

### Risk Models

Concordance with 8 PATH Statement criteria related to risk modeling was above 60% for 5 of 8 items (eTable 6 in Supplement 1). Only 25^47,49,55,60,61,62,66,69,70,72,76,80,83,87,89,92,96,97,98,101,106,108,110,112,113^ of 48 reports^47,49,53,54,55,56,58,59,60,61,62,64,65,66,69,70,71,72,74,75,76,77,78,80,82,83,86,87,89,90,91,92,93,96,97,98,99,100,101,105,106,108,109,110,111,112,113^ (52%) with positive overall findings included a risk model. Of 31 risk modeling analyses,^47,49,55,60,61,62,66,68,69,70,72,73,76,80,83,85,87,88,89,92,95,96,97,98,101,103,106,108,110,112,113^ 14^49,55,60,62,70,83,88,89,95,97,98,101,112,113^ used an external prediction model. Half of reports^47,49,55,60,62,69,70,80,83,85,88,89,95,96,97,112^ presented risk model scores by treatment group. All but 1 report^103^ presented absolute treatment effects by level of risk, and most reported relative treatment effects as well.

**Figure 2.**
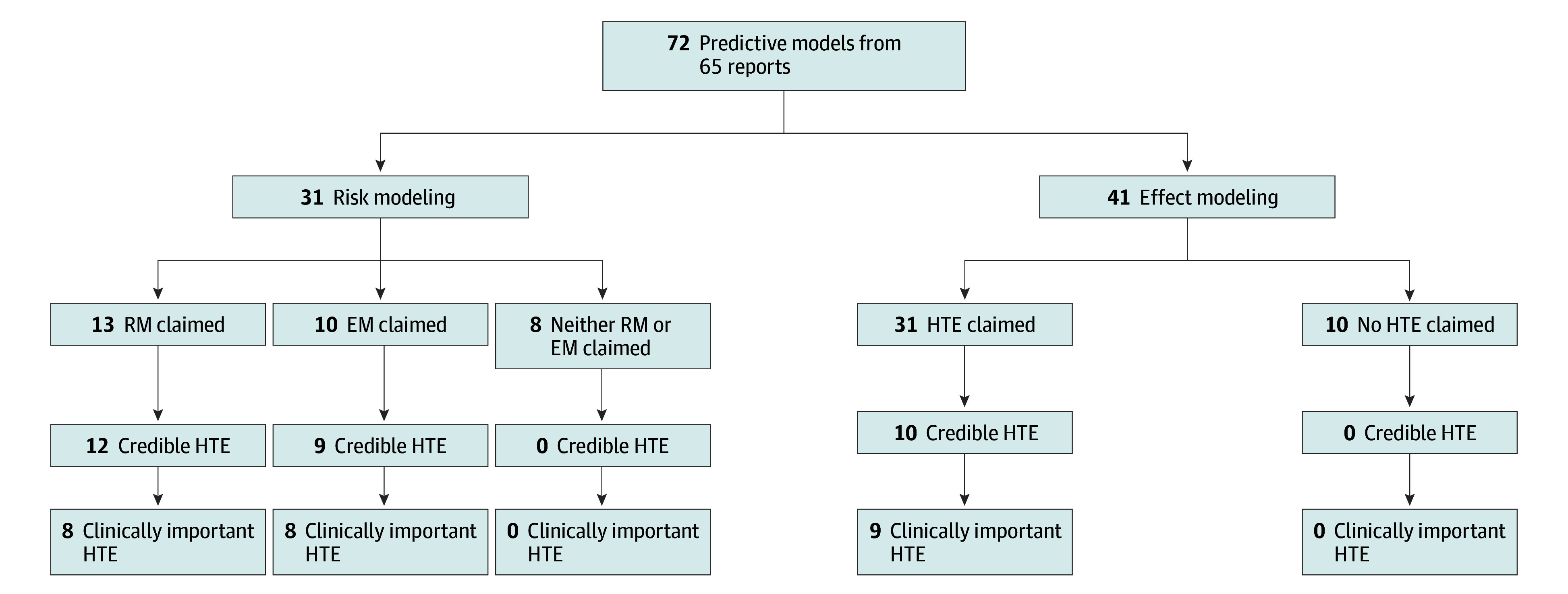
Adjudicated Results of Review of Eligible Reports Type of predictive modeling (risk or effect), claims by authors of heterogeneity of treatment effects (HTE), assessed credibility of HTE (using adapted Instrument to assess Credibility of Effect Modification Analyses criteria), and assessed clinical importance of HTE (using Predictive Approaches to Treatment effect Heterogeneity Statement definition of clinical importance). EM indicates effect modification; RM, risk modification.

Study authors claimed findings of HTE in 23 of 31 risk modeling analyses (Figure 2; eTable 7 in Supplement 1). For 13 reports,^55,61,72,76,83,87,89,95,96,98,110,112,113^ HTE was found on the absolute but not the relative scale (ie, risk magnification). For the remaining 10 reports,^47,49,60,62,66,70,80,85,92,97^ relative treatment effects also appeared to vary across levels of baseline risk. In 5 reports,^49,62,80,92,97^ relative treatment effects were greater in individuals at higher risk for experiencing trial outcomes. Relative treatment benefit was confined to individuals in the middle of the risk distribution in 3 reports^47,66,70^ or to those at lowest risk in 2 reports.^60,85^

Six reports presented risk modeling in RCTs with overall null findings.^68,73,85,88,95,103^ Only 1 report^95^ found risk magnification; 1 report^85^ found HTE on the relative scale owing to the presence of a strong effect modifier in the model.

### Effect Models

Concordance was low for 3 of the 6 PATH Statement criteria among effect modeling reports (eTable 8 in Supplement 1). Only 7^53,61,85,87,100,109,110^ of 41 reports^52,53,54,56,57,58,59,61,63,64,65,67,71,74,75,77,78,79,81,82,84,85,86,87,90,91,92,93,94,99,100,102,104,105,106,107,109,110,111,112^ restricted analyses to small numbers of covariates with strong prior evidence for effect modification. Most explored many candidate effect modifiers with little prior evidence. More than 80% of reports used recommended statistical methods, including shrinkage and internal cross-validation to reduce risks of over-fitting, but only 10 reports^52,53,54,56,71,81,93,94,109,112^ applied model findings to external datasets for validation. Authors claimed HTE in 31^52,53,54,56,57,58,64,65,67,71,74,75,77,79,81,84,85,86,90,91,92,93,94,99,100,102,104,105,107,109,112^ of 41 effect model reports (Figure 2; eTable 7 in Supplement 1). In 19^53,54,56,61,71,74,81,86,93,94,102,104,105,106,109,110,111,112^ of these 31 reports, authors heeded recommendations to report model performance metrics that evaluate prediction of individual treatment effects rather than prediction of risk for the outcome.

Twelve reports^52,57,63,79,67,81,84,85,94,102,104,107^ presented effect modeling in RCTs with overall null findings. Of these, all but 1 report^63^ claimed to have identified HTE, which was found to be credible for 3 reports.^52,81,94^

### Assessment for Credibility of HTE

Details of scoring for the 54 reports claiming HTE are given in eTable 7 in Supplement 1. Risk models were more likely to be scored as credible when ICEMAN criteria were applied (Figure 2). Twelve^55,61,72,76,83,89,95,96,98,110,112,113^ of 13 analyses claiming risk magnification and 8^47,49,60,62,70,80,85,97^ of 10 reports that found HTE on a relative scale were scored as credible. In 31 effect modeling reports, only 10 reports^52,53,56,71,81,93,94,100,109,112^ were judged credible. Three^53,100,109^ of these 10 reports considered only a small number of candidate effect modifiers with strong prior evidence. Nine^52,53,56,71,81,93,94,109,112^ of 10 reports validated effect model predictions in independent external cohorts, usually another RCT.

### Assessment for Clinically Important HTE

Reviewers judged findings from all but 6 reports^56,76,80,89,96,113^ with credible HTE to also be clinically important (Figure 2). Details for these 24 reports with clinical importance, including rationales for classification as clinically important, are summarized in Table 2. In 19 reports, RCT findings had suggested a moderate (5 reports^49,60,72,93,100^) or strong (14 reports^47,53,55,61,62,70,71,83,97,98,109,110,112^) benefit of one treatment vs another. Yet, predictive modeling identified subgroups representing 5% to 67% of the trial population for whom no benefit or possible net harm would be expected from that treatment. In 5 reports,^52,81,85,94,95^ overall results suggested no differences in outcomes between study groups, but predictive modeling identified subgroups representing 25% to 67% of participants who appeared to benefit from one treatment vs another. Findings of 6 reports with credible HTE were judged not clinically important either because the heterogeneity, although credible, did not span a threshold suggesting differing treatment choices,^96,113^ findings conflicted across outcomes,^76,89^ findings did not add clinical value to previous risk-based selection strategies,^80^ or concurrence with authors that additional investigation, possibly testing additional effect modifiers, was needed.^56^

**Table 2.** Studies Found to Have Credible and Clinically Important HTE

Reference	Clinical Condition	Outcomes	Randomized intervention	Overall RCT findings	Methods	Type of HTE identified	Clinically important subgroup differences
**Risk models (n = 15)**							
Redelmeier et al, 2020^47^ and 2022^48^	Congestive heart failure	All-cause mortality	ICD vs medical management	Strong benefit in favor of ICD (OR, 0.69)	Single RCT reanalysis; internal risk model	Relative as well as absolute effect heterogeneity, *P* < .001 for multiplicative interaction	Despite a strong overall benefit of ICD, the mortality benefit was largely confined to patients in midrange of risk (third and fourth quintiles). Intervention associated with high costs and patient discomfort
Chalkou et al, 2021^49^ and 2024^50^	MS	Relapse of MS	3 Immunologic therapies (DF, GA, and natalizumab) vs placebo	Strong benefit in favor of natalizumab; OR vs placebo: DF, 0.43; GA, 0.53; Natalizumab: 0.28	IPDMA: 3 RCTs, using network meta-analysis; external risk model	Probable relative as well as absolute HTE; test for multiplicative interaction not reported	Despite strong overall benefit of natalizumab, patients with baseline risk <30% (25% of trial population) had negligible added benefit of natalizumab vs DF. Natalizumab was associated with rare but possibly fatal complication, PML
Kumar et al,^55^ 2020	Ambulatory patients with cancer	VTE	Apixaban vs placebo	Strong benefit in favor of apixaban (aHR, 0.49)	Single RCT reanalysis; external risk model	Probable relative as well as absolute HTE; test for multiplicative interaction not reported	Despite a strong overall benefit of apixaban, patients with a baseline risk for VTE <8% (67% of the trial population) derived no benefit but experienced an excess of overall bleeding events on treatment with apixaban
Bress et al,^61^ 2021	Systolic hypertension and increased cardiovascular risk	CVD events; all-cause mortality	Intensive vs standard SBP control	Strong benefit in favor of intensive SBP control (HR for CVD event, 0.75; HR for mortality, 0.73	Single RCT reanalysis; internal risk model	Absolute but not relative HTE (risk magnification)	Despite the strong overall benefit of intensive SBP control, patients in lowest risk quartile would expect very little benefit from intensive control, despite increased costs, burden and adverse effects
Rysavy et al,^60^ 2021	Extreme prematurity	Broncho-pulmonary dysplasia	Vitamin A vs sham injection	Weak benefit in favor of vitamin A (RR, 0.89)	Single RCT reanalysis; external risk model	Relative as well as absolute HTE, *P* = .03 for multiplicative interaction	With a weak overall benefit of vitamin A therapy, predictive modeling found a strong benefit in the 50% of infants at lower predicted risk; no suggestion of benefit among infants in highest quarter of predicted risk
Kent et al,^62^ 2021	PFO-associated stroke	Recurrent stroke	Percutaneous PFO closure vs medical therapy	Strong benefit in favor of PFO closure (aHR, 0.41)	IPDMA of 6 RCTs; external risk model	Relative as well as absolute HTE, *P* = .003 for multiplicative interaction	Despite a strong overall benefit, analyses identified a subgroup of 15% of trial population unlikely to have had a PFO-related stroke. This subgroup received no benefit from PFO closure and were at higher risk of procedure-related complications
Taylor et al,^70^ 2022	Hospitalized patients with sepsis	30-d Mortality and readmission	Nurse-navigator led Sepsis Transition and Recovery intervention vs usual care	Weak benefit in favor of the intervention (aOR, 0.80)	Single RCT reanalysis; external risk model	Relative as well as absolute effect heterogeneity; 95% CIs for quartile-specific ORs do not overlap	Despite findings of a modest overall benefit of the program, the benefit was confined to patients in the midrange of predicted risk (middle 2 quartiles). Intervention is associated with greater costs
Gencer et al,^72^ 2022	AF	Net composite of stroke/systemic embolism, major bleed, all-cause death	Lower vs higher dose regimens of edoxaban vs warfarin	Weak benefit for either dose of edoxaban vs warfarin (HR for lower, 0.83; HR for higher, 0.89)	Single RCT reanalysis; risk stratification based on count of number of high-risk features	Absolute HTE (*P* = .001) but probably not relative HTE (*P* = .07) (risk magnification)	With a weak overall effect, the absolute benefits of either dose of edoxaban vs warfarin were negligible in patients with 0-1 risk factors but increased for both stroke/embolism and major bleeding end points as number of risk factors increased. May not justify switching in low-risk patients well managed on warfarin
Trinks-Roerdink,^83^ 2023	AF	All-cause mortality	Integrated atrial fibrillation care vs usual care	Strong benefit of integrated care (aHR, 0.55)	Single RCT reanalysis, external risk model	Absolute but no relative HTE (*P* = .93 for multiplicative interaction) (risk magnification)	Despite a strong overall benefit of the program, benefits were shown to be predominantly in persons in highest 25% of risk distribution; minimal benefit with increased costs in lower 75%
Goligher et al,^85^ 2023	Hospitalized SARS-CoV-2 infection	Organ support–free days; hospital survival	Therapeutic-dose heparin vs usual pharmacologic thromboprophylaxis	No overall benefit in population (OR for benefit, 1.05)	IPDMA of 3 trials; reanalysis; subgroup analyses, internal risk model and effect model	Apparent qualitative interaction on both relative and absolute scales; statistical significance not tested for risk model	Although no overall benefit was observed, a clear benefit was shown for the 60% of persons in lower deciles of risk score (not requiring organ support at baseline); apparent harm for those needing intensive care at baseline (highest 3 deciles of risk score); note: similar HTE was suggested in subgroup analyses and in an effect model
Xu et al,^95^ 2024	Healthy people age ≥70 years	Composite of death, dementia, or disability	Low-dose (100 mg) aspirin vs placebo	Null (HR, 1.01; 95% CI, 0.92-1.11)	Single RCT reanalysis, external risk model	Absolute HTE (*P* = .03) but no relative effect heterogeneity (risk magnification)	Despite a null overall effect, risk model showed that highest-risk 20% of patients benefitted (ARR = 15.1%; 95% CI, 4.0%-26.3%), with little suggestion of any benefit in remaining 80%
Paules et al,^97^ 2024	Patients hospitalized with COVID-19	28-d Mortality; progression to mechanical ventilation or death; recovery rate	Baricitinab + remdesivir vs remdesivir alone	Strong benefit of combination for each outcome (HR for death, 0.65; 95% CI, 0.39-1.09)	Single RCT reanalysis, external risk model	Clear heterogeneity on absolute scale for all 3 outcomes (risk magnification); also, possible relative effect heterogeneity for the time-to-recovery outcome, with HR for the highest risk quartile significantly greater than for lowest risk quartile	A strong overall benefit of baricitinib in the trial was due to a very strong benefit on absolute and relative scales in the highest risk quartile, and likely the second-highest risk quartile; with little apparent effect in the lower 50% of predicted risk
Vickers et al,^98^ 2024	Localized prostate cancer	15-y Prostate cancer mortality	Radical prostatectomy vs conservative care	Strong benefit of surgery (HR, 0.55)	Separate RCT reanalyses from 2 trials, external risk models	Established that HRs were constant across levels of baseline risk (*P* for interaction on relative scale >.20 in both trials); therefore, risk magnification with large differences in predicted benefit across levels of baseline risk	Despite a strong overall relative benefit, absolute benefit of radical prostatectomy in the lowest risk quartile of current cohorts would be approximately a 0.2% reduction in 15-y mortality. For higher-risk subgroups, benefit is substantial. This demonstration of risk magnification is useful information for shared decision-making
De Winkel et al,^110^ 2024	Acute subarachnoid hemorrhage	Functional status at 2 mo; retreatment or rebleed by 10 y	Endovascular coiling vs intracranial clip	Strong benefit of coiling for good functional status (OR for benefit, 1.5; 95% CI, 1.3-1.7); but a strong benefit of clipping for 10-y treatment failure: (HR, 0.30; 95% CI, 0.2-0.4)	Single RCT reanalysis; ordinal regression for functional status; proportional hazards model for treatment failure; began as exploratory effect modeling but reduced to risk modeling	Established strong risk magnification favoring intracranial clip for outcome of retreatment or rebleed. Modest differences in absolute benefits of coiling across range of risk for poor functional outcome	Despite competing overall findings for the 2 outcomes indicating need for shared decision-making, a subgroup of 6% of patients were predicted to have a strong benefit of clipping for the rebleed/retreatment outcome with very low risk and negligible (<2%) differences between treatments for the functional status outcome
Smit et al,^112^ 2023	Community-acquired pneumonia	30-d Mortality	Corticosteroids vs placebo	Strong benefit of corticosteroids (OR, 0.72; 95% CI, 0.56-0.94)	IPDMA of 6 trials, external risk models; also examined an effect model	No evidence of treatment-risk interactions for relative effects, but moderate risk magnification	Despite a strong overall benefit of corticosteroids, the risk model identified 36% of patients with low risk who would have almost no predicted benefit (and increased corticosteroid-related risks for rehospitalization and hyperglycemia)
**Effect models (n = 9)**							
Park et al,^52^ 2022	Hospitalized SARS-CoV-2 infection without mechanical ventilation	Ordinal COVID-19 clinical status scale	CCP vs control	Null (no overall association between CCP and patient outcomes)	IPDMA of 8 trials; proportional odds model; external validation in multiple datasets	Relative treatment effect differences demonstrated with nonoverlapping 95% CIs	Despite null findings in overall trial population, predictive modeling identified 3 approximately equal-sized subgroups, 1 with high benefit from CCP, 1 with modest benefit from CCP, and 1 with modest harm from CCP
Takahashi et al,^53^ 2020	De novo 3-vessel and left main coronary artery disease	5-y MACE and 10-y all-cause death	CABG vs PCI	Strong benefit in favor of CABG (HR for 10-y mortality, 0.84; HR for 5-y MACE, 0.78)	Single RCT reanalysis; Cox proportional hazards regression; external validation in IPDMA of 3 trials	Multiplicative interactions of treatment choice with 2 prespecified effect modifiers	Predictive model identifies half of patients who clearly benefit from CABG (vs PCI); and half in whom there is no expectation of greater benefit with the more invasive procedure for either 5-y MACE or 10-y all-cause death
Dennis et al,^71^ 2022	Type 2 diabetes	Hb A_1c_ at 6 mo postinitiation	Initiation of SGLT-2 inhibitors vs DPP-4 inhibitor as add-on therapy	Weak benefit in favor of SGLT-2 inhibitor: mean difference in HbA_1c_, 12 mmol/mol^a^	Model built in large observational cohort; linear regression; external validation in multiple RCTs	Clear subgroup heterogeneity in absolute differences in treatment effects on HbA_1c_ control	Predictive model identifies a large subgroup, comprising 60% of trial participants, with strong benefit with SGLT-2 inhibitor (vs DPP-4 inhibitor); a small group (approximately 5%) do better if a DPP-4 inhibitor is initiated
Seitz et al,^81^ 2023	Respiratory distress	Successful intubation on the first attempt	Bougie vs stylet	Null (nonsignificant 6.8–percentage point difference in favor of stylet)	Single RCT reanalysis; causal forest; external validation in nonrandom subset	Absolute and relative effect differences (*P* = .02 for multiplicative interaction of treatment with predicted treatment effect)	Despite null findings in the overall trial population, predictive modeling identifies 25% of patients in the validation cohort who benefit from use of the stylet (vs bougie); 25% of patients with a possible marginally better result when bougie is used; with no treatment effect differences in the remainder
Venkatasu-bramaniam et al,^93^ 2023	Type 2 diabetes	Hb A_1c_ reduction at 6 mo	SGLT-2 inhibitors vs DPP-4 inhibitors as add-on therapy	A very modest benefit of SGLT-2 inhibitors (1.6 mmol/mol^a^ greater HbA_1c_ reduction at 6 mo)	IPDMA of 2 trials; penalized linear regression and causal forest models, external validation in large observational cohort (n = 18 741)	Both penalized regression and causal forest models indicated significant HTE (*P* = .005 for causal forest); significance testing not reported for regression model, but benefit prediction in external cohort was excellent	Regression model reliably identified a subgroup of 87% of validation cohort who did better with an SGLT-2 inhibitor, including 30% of all patients who were predicted to have a >5 mmol/mol^a^ larger reduction in HbA_1c_ with the SLGT-2 inhibitor. For 13% of patients, HbA_1c_ reduction was predicted to be better with DPP-4 inhibitors
Buell et al,^94^ 2024	Patients requiring mechanical ventilation	28-d Mortality	Lower vs higher o_2_ saturation targets	Null findings in multiple trials of moderate size	Single RCT reanalysis; ensemble machine learning effect models (RBoost); external validation in a second RCT	Both relative and absolute HTE (*P* for interaction = .02) shown across tertiles of predicted benefit in the validation trial	Null overall findings in 7 prior RCTs comparing lower vs higher o_2_ saturation targets trials; these validated analyses identified subgroups of patients who clearly benefited from either lower (33% of all patients) or higher o_2_ saturation targets and also identified the most critical individual characteristics for predicting benefit
Arnold et al,^100^ 2024	Patients with chronic coronary artery disease	Angina symptoms at 12-mo follow-up	Coronary revascularization vs medical therapy	A modest benefit of revascularization for angina symptoms	Single RCT reanalysis; ordinal proportional odds regression effect model	Strong absolute HTE (*P* < .01) for 1 baseline covariate (symptom severity)	Weak overall benefit of revascularization; effect model identified 1 strong effect modifier and subgroups of 75% in whom the predicted benefit was strong, and 25% predicted to experience little if any benefit
Ninomiya et al,^109^ 2023	Patients with de novo 3-vessel or left main coronary artery disease	5-y MACE and 10-y all-cause death	CABG vs PCI	Strong benefit in favor of CABG (HR for 10-y mortality, 0.84; HR for 5-y MACE = 0.78)	Single RCT reanalysis; proportional hazards regression effect model with 2 prespecified interactions; external validation in observational cohort	Strong absolute and relative scale HTE (*P* < .005 for 2 covariates)	Despite relatively strong overall benefit of CABG, effect model identifies 25% of patients who would have better predicted outcomes with PCI, 50% with very strong benefit of CABG, and 25% for whom there is little predicted difference in benefits
Smit et al 2020^112^ 2023	Community-acquired pneumonia	30-d Mortality	Corticosteroids vs placebo	Strong benefit of corticosteroids (OR 0.72; 95% CI, 0.56-0.94)	IPDMA of 6 trials; penalized logistic regression–based effect models; external validation in 2 addition RCTs	Strong HTE driven primarily by interaction with 1 baseline covariate(C-reactive protein level) (*P* = .05)	Despite a relatively strong overall benefit of corticosteroids, the effect model identified nearly half of patients, with lower levels of C-reactive protein, with no predicted benefit from corticosteroids, and experienced adverse effects, including rehospitalization and hyperglycemia

Abbreviations: AF, atrial fibrillation; aHR, adjusted hazard ratio; aOR, adjusted odds ratio; ARR, absolute risk reduction; CABG, coronary artery bypass graft; CCP, convalescent plasma; CVD, cardiovascular disease; DF, dimethyl fumarate; DPP-4, dipeptidyl peptidase-4; GA, glatiramer acetate; HbA_1c_, hemoglobin A_1c_; HTE, heterogeneity of treatment effect; ICD, implantable defibrillator; IPDMA, individual patient data meta-analysis; MACE, major adverse cardiac events; MS, multiple sclerosis; o_2_, oxygen; PCI, percutaneous coronary intervention; PFO, patent foramen ovale; PML, progressive multifocal leukoencephalopathy; RCT, randomized clinical trial; RR, relative risk; SBP, systolic blood pressure; SGLT-2, sodium-glucose cotransporter 2; VTE, venous thromboembolism.

^a^ To convert to percentage of total hemoglobin, multiply by 2.2.

## Discussion

This scoping review found that in the 4.5 years following publication of the PATH Statement, a steadily increasing number of publications across a wide range of clinical areas has cited the PATH Statement and used predictive modeling to examine possible HTE in RCT results. Among the 65 reports we identified, more than one-third (24 reports [37%]) found HTE that was both credible when ICEMAN criteria were applied and clinically important, suggesting that patients and clinicians could often do better than relying solely on mean effects.

Risk modeling was more likely than effect modeling to produce findings of credible HTE in positive RCTs because of its relative simplicity and strong clinical and theoretical rationale. Importantly, HTE in risk models was not always confined to the absolute scale (risk magnification). In 8 reports, credible and important HTE was identified on the relative scale as well. In 4 reports,^49,55,62,97^ relative as well as absolute effects were greater for persons at higher predicted risk. In 2 reports,^60,85^ persons at low risk benefited while patients at high risk may have been harmed by the same treatment; in 2 reports,^48,70^ maximal benefit was found for those in the middle range of risk. This U-shaped, or “sweet spot,” pattern^48^ is clinically intuitive and has also been observed elsewhere.^116^

These findings of HTE on the relative scale demonstrate that a simple assumption of risk magnification is not always confirmed and suggest the importance of routine risk modeling in RCTs when overall results are positive. Such findings may also provide initial insights about specific effect modifiers. Traits incorporated into risk scores as predictors of study outcomes may sometimes also be treatment effect modifiers on the relative scale, either directly or as proxies for unmeasured attributes. In an RCT comparing therapeutic-dose heparin with usual thromboprophylaxis for patients hospitalized with COVID-19,^85^ respiratory status at baseline was the most potent predictor of risk for poor outcomes but was also found to be a strong modifier of heparin’s treatment effect in initial subgroup analyses. Only patients with better baseline respiratory status benefited from heparin. When respiratory status was incorporated into a risk prediction score, only patients with lower scores appeared to benefit. In 3 RCTs^48,60,70^ where incidence of study outcomes was particularly high (range, 27%-61%), no benefit was observed in the highest stratum of predicted risk. For such individuals with extremely high risk, risk prediction models likely included attributes reflecting irreversible disease or competing causes of the outcome that would make treatment futile.

Findings of HTE on the relative scale in risk modeling may provide a rationale for moving to effect modeling to improve on individual treatment effect estimation^29^ and possibly to identify specific, strong effect modifiers. Effect modeling may also have utility in examining RCTs with overall null findings, where risk modeling lacks a rationale. In both situations, need for larger sample sizes and external populations for validation, as well as the PATH Statement’s general caution regarding searches for HTE in negative trials should be kept in mind.

Despite citing the PATH Statement, many authors nevertheless chose to use only effect modeling, even when overall RCT findings were positive. Several authors^59,77,89^ expressed concerns that although risk modeling can create patient subgroups well-matched on risk, subgroup members may still be heterogenous for the specific characteristics that contributed to their risk scores or for other treatment effect–modifying characteristics not related to overall risk. If so, risk modeling could potentially miss important HTE or incorrectly specify predictions of individual treatment effects.

Seven reports^61,85,87,92,106,110,112^ presented both risk and effect modeling of the same RCT data. Effect modeling added new insights to risk modeling in only 1 report.^112^ In this individual participant data meta-analysis of 8 RCTs of corticosteroids for community-acquired pneumonia,^112^ effect modeling identified a single powerful relative treatment effect modifier, C-reactive protein, a variable that was not included in the risk model. Although both models found credible HTE, the effect model performed better in external validation. More generally, the existence of strong effect modifiers, whether known in advance or not, is a likely prerequisite for finding that an effect model improves on risk modeling.^29^

Nearly all effect modeling reports followed PATH Statement suggestions to use shrinkage methods and internal validation strategies to reduce over-fitting. Nevertheless, inconsistencies within and between reports illustrated the persistent challenge of false-positive signals of HTE in exploratory effect modeling and underscore the critical value of external validation when apparent HTE is found. For example, 2 reports^59,86^ applied causal forest algorithms to data from the SPRINT and Action to Control Cardiovascular Risk in Diabetes trials evaluating intensive systolic blood pressure control. One report^59^ found evidence of HTE, the other did not. In 2 reports^64,82^ from a trial of dabigatran vs warfarin for stroke prevention in atrial fibrillation, 1 report^64^ suggested interactions of 3 covariates with treatment choice and significant HTE; the second, using 4 machine learning algorithms applied to the same RCT data, found no evidence for HTE. Two reports^63,111^ compared results of multiple machine learning algorithms, finding inconsistent evidence for HTE between algorithms and even within algorithms when random initiation seeds were altered.^63^ Two reports^93,112^ compared regression-based methods with machine learning algorithms in effect modeling, and both found that regression models performed better in external validations.

The inconsistency of exploratory machine learning approaches to effect modeling has also been addressed by others.^20^ Most effect models reviewed here considered large numbers of candidate treatment interactions despite relatively modest numbers of outcome events. Specification of optimal methods in the rapidly evolving area of data-driven effect modeling is beyond the scope of this review. Greater experience with newer methods might inform more credible and reproducible results than found in our review. Larger databases and ready access to all data when redundant RCTs have addressed the same question will be important for optimally identifying HTE. External validation, as shown in this review, and generalization of findings to other populations^117^ will likely continue to be critical in establishing credibility.

Given the frequent utility of risk modeling, sponsors of new RCTs should consider in advance whether appropriate external risk models exist and plan for collection of baseline data needed for assigning or estimating individual risk. Within the past decade, editorial guidelines for reporting RCTs have come to require presentation of absolute as well as relative treatment effects because of their greater relevance in clinical decision-making.^118,119,120^ We suggest that reports of positive RCTs could be further enhanced by requiring that treatment effects, in both relative and absolute terms, be presented in relation to baseline risk. Ultimately, it will remain critical to demonstrate the safety and effectiveness of any predictive model when used for personalizing treatment choices in clinical populations.

### Limitations

This study has some limitations. Our search strategy does not capture all reports of predictive modeling for HTE and, given the prominence of risk modeling in the PATH Statement, may preferentially identify risk models rather than effect models. Nevertheless, effect models, including those using flexible machine learning methods, were well represented within our large study sample, accounting for well over half the models we identified. We attempted broader searches for reports of predictive models in RCTs using keyword and title/abstract terms in PubMed for the same time period. We identified only 7 eligible reports in total, 3 of which were included in our review. The low yield of these searches illustrates the challenge in finding an alternative search strategy.

It is also possible that reporting and publication bias may have affected our findings. Analyses that did not identify clinically meaningful findings or models that failed to validate may be less likely to be published, potentially skewing the sample (of both risk and effect models) toward more credible and clinically important results. Despite these potential biases, we believe that the large number of reports found using the present search strategy offers a valid and relevant approach for assessing the PATH Statement’s influence. Additionally, we acknowledge that some subjectivity remains in applying the ICEMAN criteria and the close association of 2 authors (J.V.S. and D.M.K.) with production of the PATH Statement should be kept in mind.

## Conclusions

In this scoping review, we observed an increasing frequency of reports presenting multivariate predictive models of HTE in RCT data in the years following publication of the PATH Statement. Risk modeling provides a straightforward and clinically relevant initial approach when overall trial findings are positive and often identified credible, clinically important HTE in this review. More precise predictions of individual treatment effects may be obtainable using effect models, especially if strong effect modifiers are known or suspected. Because most effect modeling reports reviewed here were highly exploratory, external validation was important in strengthening the credibility of HTE findings.

## Notes

Supplement 1. **eTable 1.** PATH Statement Consensus Criteria **eTable 2.** Electronic Search Strategy and 16 Reports Excluded from Review Because They Do Not Meet PATH Definition of a Predictive Model **eTable 3.** Variables Collected, Possible Values, Initial Disagreement Rates for Items Doubly Reviewed **eTable 4.** PATH Follow-Up Study Review Coding Instructions **eTable 5.** Coding Instructions for Applying Four ICEMAN Criteria to Assess Credibility of HTE and Summarizing the Four Criteria Responses Into an Overall Credibility Score **eTable 6.** Consistency of Report Methods With PATH Statement Criteria for Risk-Modeling Analyses **eTable 7.** Evaluation of Claimed HTE Using Four Criteria Adapted From ICEMAN to Assess Overall Credibility of HTE and Clinical Importance **eTable 8.** Consistency of Report Methods With PATH Statement Criteria for Effect-Modeling Analyses

Supplement 2.
Data Sharing Statement
