# Supplementary material for: Predictive Modeling of Heterogeneous Treatment Effects in RCTs: A Scoping Review
Source: JAMA Netw Open. 2025 Jul 22;8(7):e2522390. doi: 10.1001/jamanetworkopen.2025.22390 (PMC12284745; doi:10.1001/jamanetworkopen.2025.22390)
Supplement: Supplement 2. — Data Sharing Statement [file jamanetwopen-e2522390-s002.pdf]

Data Sharing Statement

Selby. Predictive Modeling of Heterogeneous Treatment Effects in RCTs. *JAMA Netw Open*.
Published July 22, 2025. doi:10.1001/jamanetworkopen.2025.22390

Data
Data available: Yes
Data types: Other (please specify)
Additional Information: meta-data on the 65 reports reviewed; the data dictionary for these data is included in the supplemental materials.
How to access data: Excel spreadsheet of all review data collected may be obtained from first author, Joe V Selby, at jvselby@outlook.com
When available: With publication

Supporting Documents
Document types: Other (please specify)
Additional Information: data dictionary and review instructions are included in the online supplemental materials submitted for review.
How to access documents: Contact the first author, Joe V Selby, by email at jvselby@outlook.com
When available: With publication

Additional Information
Who can access the data: resaerchers whose proposed use has been reviewed and approved
Types of analyses: for any purpose
Mechanisms of data availability: after approval of proposal and with a signed data access agreemen
Any additional restrictions: none
